# Continuous succinic acid fermentation by *Actinobacillus succinogenes* in a packed-bed biofilm reactor

**DOI:** 10.1186/s13068-018-1143-7

**Published:** 2018-05-14

**Authors:** Mariateresa Ferone, Francesca Raganati, Alessia Ercole, Giuseppe Olivieri, Piero Salatino, Antonio Marzocchella

**Affiliations:** 1 Dipartimento di Ingegneria Chimica, dei Materiali e della Produzione Industriale, Università degli Studi di Napoli Federico II, P.le V. Tecchio 80, 80125 Naples, Italy; 2 Wageningen University and Research Centre, Droevendaalsesteeg 1, P.O. Box 8129, 6708 PB Wageningen, The Netherlands

**Keywords:** Biorefinery, Succinic acid, *Actinobacillus succinogenes*, Biofilm, Lignocellulose

## Abstract

**Background:**

Succinic acid is one of the most interesting platform chemicals that can be produced in a biorefinery approach. In this study, continuous succinic acid production by *Actinobacillus succinogenes* fermentation in a packed-bed biofilm reactor (PBBR) was investigated.

**Results:**

The effects of the operating conditions tested, dilution rate (D), and medium composition (mixture of glucose, xylose, and arabinose—that simulate the composition of a lignocellulosic hydrolysate)—on the PBBR performances were investigated. The maximum succinic acid productivity of 35.0 g L^−1^ h^−1^ and the maximum SA concentration were achieved at a *D* = 1.9 h^−1^. The effect of HMF and furfural on succinic acid production was also investigated. HMF resulted to reduce succinic acid production by 22.6%, while furfural caused a reduction of 16% in SA production at the same dilution rate.

**Conclusion:**

Succinic acid production by *A. succinogenes* fermentation in a packed-bed reactor (PBBR) was successfully carried out for more than 5 months. The optimal results were obtained at the dilution rate 0.5 h^−1^: 43.0 g L^−1^ of succinic acid were produced, glucose conversion was 88%; and the volumetric productivity was 22 g L^−1^ h^−1^.

**Electronic supplementary material:**

The online version of this article (10.1186/s13068-018-1143-7) contains supplementary material, which is available to authorized users.

## Background

Sustainable production of chemicals and fuels from renewable resources is a priority for the modern societies. Indeed, the growing awareness of the environmental impact of petrochemical processes has increased the interest for alternative routes for sustainable productions of commodities. According to this scenario, biorefineries offer an excellent opportunity to replace the oil refinery with the bio-based-derived products [1].

Organic acids—in particular bicarboxylic acids—are expected to play a key role in the feasibility of future biorefineries because of their huge potential as platform molecules. Succinic acid (SA)—a four carbon bicarboxylic acid produced as an intermediate in the tricarboxylic acid (TCA) cycle—is a very interesting bicarboxylic acid that can be produced by fermentation of renewable resources. The high potential of the SA has been pointed out by the US Department of Energy that included it among the 12 top value-added chemical produced from biomass [2]. Indeed, the SA is currently used in the food industry, as a pH regulator and as a flavoring agent, in the pharmaceutical industry, as additive for the preparation of drugs, in the agricultural food and as ion chelator and surfactants [3]. Because of its structure, SA can be also used as a building block chemical and converted to 1,4-butanediol, *γ*-butyrolactone, *N*-methyl-2-pyrrolidone, tetrahydrofuran, 2-pyrrolidone, maleic acid and maleic anhydride, polyammides, and polyesters [4]. The industrial success of the SA produced via the biotechnological route depends on the production cost. The current price of succinic acid produced via the petrochemical route is about 2.94 $/kg [5] and any efforts should be addressed to reduce the production cost around 1$/kg to propose the bio-SA as a potential alternative to the chemical route, as it is required for the production of commodity products by the chemical industry [6]. Several companies—such as BioAmber, Myriant, Succinity, and Reverdia—have developed processes for the production of bio-succinic acid by proprietary microorganisms and strain. However, the current commercial production of SA is based on the use of pure sugars derived from starch-based raw materials that potentially compete with food resources [7].

The key issues for the success of industrial processes for the production of succinic acid via the biotechnological route include the selection/development of an SA producing microorganism, the selection of the feedstock, the specific productivity of the fermenters, and the development of an efficient recovery process for SA.

A potential microbial platform to produce SA is *Actinobacillus succinogenes*: a microorganism characterized by the best bench-scale performances [8–10]. It has been pointed out that these bacteria can produce SA at high yields and concentration during mixed-acid, batch fermentation, using a variety of carbon source [11–13]. However, according to the previous studies [14, 15], *A. succinogenes* growth is inhibited by the acids produced during the fermentation and this feature reduces the volumetric productivity of batch processes and increases the dead times. These drawbacks can be addressed by the use of single culture biofilm reactors; the main advantages of this typology of reactors include high cell density achieved, operability at high dilution rate without cell washout, high specific productivity, and the possibility to reuse the biofilm support [16]. Moreover, biofilms are known for their stability to long-term continuous operation and the enhanced tolerance to toxic compounds [17, 18].

In addition to the choice of the biocatalyst and of the reactor type, the feedstock selection plays a key role in the economics of the process [19]. Lignocellulosic biomass is generally considered an ideal feedstock for the production of bio-products because of their low cost, high availability, and un-competitiveness with food sources [20, 21]. Pretreatment of lignocellulosic biomass should be carried out via combined thermo-chemical and enzymatic treatment to produce C5 and C6 sugars; however, microbial inhibitors—such as furfural, 5-hydroxymethylfurfural (HMF), acetic acid, and low-molecular-weight phenolic compounds—may be produced during the pretreatment process and they reduce the performance of the SA production process.

The present contribution regards the continuous production of succinic acid by wild-type biofilm of *Actinobacillus succinogenes* in a packed-bed reactor. The performance of the reactor was assessed under a wide range of dilution rate and by feeding the reactor with streams bearing several sugars (single and mixed). The continuous fermentation process was characterized in terms of succinic acid concentration, productivity and selectivity as well as sugar conversion. In addition, the effect of two putative fermentation inhibitors (e.g., furfural and HMF) was investigated by supplementing the feeding with a single inhibitor.

## Methods

### Microorganism and media

*Actinobacillus succinogenes* DSM 22257 was supplied by DSMZ. Stock cultures were reactivated according to the procedure suggested by the supplier. Reactivated cultures were stored at − 80 °C. The thawed cells were inoculated in 15 mL Hungate tubes containing 12 mL of containing Brain Hearth Infusion broth (BHI). Cells were grown in anaerobic conditions for 24 h at 37 °C. Then, the precultures were inoculated into fermentation bottles.

The feeding medium consisted of: 5 g/L Yeast Extract (nitrogen source), 1 g L^−1^ NaCl, 0.3 g L^−1^ Na_2_HPO_4_, 1.4 g L^−1^ NaH_2_PO_4_, 1.5 g L^−1^ K_2_HPO_4_, 0.2 g L^−1^ MgCl_2_·6H_2_O, and 0.23 g L^−1^ CaCl_2_·2H_2_O. The medium was sterilized in autoclave (121 °C, 20 min). The carbon source used in the continuous test was varied according to the each experimental test campaign, as explained in “Design of experiment” section.

All the chemicals used were purchased from Sigma-Aldrich.

### Bioreactor

**Fig. 1:**
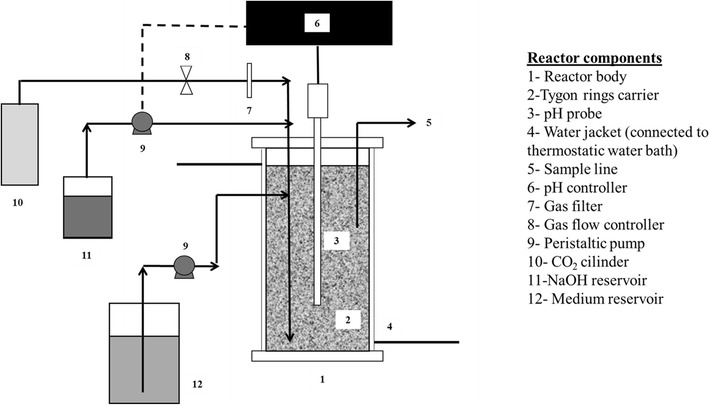
Outline of the apparatus used for continuous tests. The reactor consisted of a 166-mL glass bottle (5 cm ID, 8.5 cm high), jacketed for the heat exchange. The working reaction volume was set by means of an overflow duct

The bioreactor used for the continuous fermentation test is shown in Fig. 1. The reactor consisted of a 166-mL glass bottle (5 cm ID, 8.5 cm high), jacketed for the heat exchange. The working reaction volume was set by means of an overflow duct. Carbon dioxide was sparged at the reactor bottom to support anaerobic conditions and to provide the CO_2_ for the succinic acid production pathway. The system for pH control consisted of a pH meter, a peristaltic pump, a vessel with NaOH 0.3 M solution, and a pH controller. Temperature was controlled at 37 °C using a water jacket connected to a thermostatic water bath.

The reactor filled with the Tygon support was sterilized in autoclave at 121 °C for 20 min. The gas stream was sterilized by filtration (cutoff 0.2 μm, Millipore). The sterile medium was fed at the bottom of the reactor with a peristaltic pump.

No chemical was used to assist cell immobilization on the selected carrier [22].

### Analytical methods

Cell density was measured as optical absorbance at 660 nm (OD_660_) using a spectrophotometer (Cary-50 Varian).

The concentration of soluble species was measured in the liquid phase after spinning down the cells by centrifugation (13000*g*, 10 min). Sugar and organic acid concentrations were measured by means of a high-performance liquid chromatography (HPLC) (HP1250 working station system—Agilent Technologies, USA) equipped with a cation-exclusion column (Aminex HPX-87H; 300 mm × 7.8 mm, 9 µm; Bio-Rad Chemical Division, Richmond, CA). Analytes were detected by UV absorbance (Agilent Technologies, G1315D) and refractive index (Agilent Technologies, G1362A). H_2_SO_4_ 5 mM was used as mobile phase at 0.6 mL min^−1^ flow rate at room temperature. The injection volume was 20 µL.

### Experimental procedures and data analyses

300 µL of glycerol stock culture was transferred in 10 mL Hungate tubes containing the seed culture media (37 g L^−1^ of BHI broth). The precultures were incubated for 24 h under anaerobic batch conditions, and then, 30 mL of actively growing cells were inoculated into the reactor.

Tests aimed at succinic production were carried out with the packed-bed biofilm reactor (PBBR) operated at preset conditions. 34.4 g Tygon rings used to prepare a 4.5 cm high packed bed. The volume of the reactor was set at 40 mL by means of the overflow duct.

The start-up of the biofilm in the PBBR was carried out according to the procedure reported by Napoli et al. [23].

The dilution rate (D)—the ratio between the feeding volumetric flow rate and the volume of the fixed bed—ranged between 0.5 and 2.4 h^−1^. The biofilm reactor performances were assessed by measuring the concentration of sugar(s) and metabolites provided that the steady state had stabilized—concentration of all metabolites and sugars constant—for at least ten times the reactors mean residence time (1/D). Reactor performances were reported in terms of sugar conversion degree (*ξ*_*S*_), sugar-to-‘‘i-species’’ fractional yield coefficient (*Y*_*i*/*S*_), succinic acid productivity (*W*_SA_), and succinic acid selectivity with respect to the other acids (*χ*_SA_). *ξ*_*S*_, *Y*_*i*/*S*_, *W*_SA_, and *χ*_SA_ were assessed assuming that: (i) the feeding did not contain cells and metabolites, and (ii) the gas stripping of metabolites was negligible. According to these assumptions by means of Eqs. (1)–(4):

1 $$\xi_{S} = \frac{{S_{\text{IN}} - S_{\text{OUT}} }}{{S_{\text{OUT}} }}$$

2 $$Y_{{{\raise0.7ex\hbox{$i$} \!\mathord{\left/ {\vphantom {i S}}\right.\kern-0pt} \!\lower0.7ex\hbox{$S$}}}} = \frac{{i_{\text{OUT}} }}{{S_{\text{IN}} - S_{\text{OUT}} }}$$

3 $$W_{\text{SA}} = D \cdot {\text{SA}}_{\text{OUT}}$$

4 $$\chi_{\text{SA}} = \frac{{D \cdot {\text{SA}}_{\text{OUT}} }}{{D \cdot \left( {{\text{SA}}_{\text{OUT}} {\text{ + AA}}_{\text{OUT}} {\text{ + FA}}_{\text{OUT}} } \right)}},$$

where *S*, SA, AA, FA, and “*i*” are the concentration of sugar, succinic acid, acetic acid, formic acid, and generic metabolites, respectively, measured in the feeding (suffix IN) and in the effluent (suffix OUT).

The mass of biofilm in the reactor was assessed at the end of the run in agreement with the procedure reported in Raganati et al. [22]. Briefly, the dry carrier was weighted before filling the reactor; at the end of the test, the reactor was rinsed with sterile water to remove sugars and metabolites and the carriers with the attached biofilm were harvested and dried at 40 °C for 24 h. Finally, the dried mass of the biomass and carriers was weighted, and the dried mass of the biofilm in the reactor was assessed as the difference between the weight of the carrier biofilm and the carriers.

### Design of experiments

The tests were aimed to assess the performance of the PBBR under a wide range of dilution rate and by feeding the reactor with stream bearing a spectrum of substrate. The *D* was quasi-steadily increased in each experimental set: the *D* was increased at the new value, close to the previous one, and it was kept constant until a steady-state condition established.

The set of experiments was carried out by feeding the PBBR, with a glucose-based medium (glucose concentration set at 50 g L^−1^) and the dilution rate was set between 0.5 and 2.4 h^−1^.

The second set of experiments was aimed to adapt the cells to a xylose-based medium, the main pentose sugar present in a lignocellulosic hydrolysate. The feeding was a solution of glucose and xylose at percentage of xylose progressively increased from 0 up to 100%. The total sugar concentration (glucose + xylose) in the feeding was set at 50 g L^−1^. The dilution rate was set at 1.24 h^−1^.

After evaluating how the PBBR performances changed increasing the xylose concentration in the media, the effect of the dilution rate on the succinic acid production by using a xylose-based medium was investigated. For this set of experiments, the xylose concentration was set to 40 g L^−1^ and the dilution rate was ranged between 0.5 and 1.44 h^−1^.

The fourth set of experiments was carried out by feeding the PBBR with a synthetic medium that mime the composition of a lignocellulosic hydrolysate (inhibitor-free). The feeding was a solution of glucose, arabinose and xylose (GAX): the total sugar concentration was set at 80 g L^−1^ and the mass ratio between the sugars was set to at 55:15:30 [24]. The dilution rate was set between 0.7 and 1.44 h^−1^.

A *D*-jumping strategy was adopted to assess the repeatability of the biofilm reactor performance with respect to the dilution rate. Provided that the reactor steady-state at the prefixed value of *D* = D^*^, the feeding stream rate was changed to operate the reactor at a *D* equal to a fraction of the previous one (say *D*^+^) and close to a *D* value already investigated. The performances of the reactor were measured until the new steady-state condition established and they were compared with those measured under the previous *D*^+^. The comparison of the performances of the biofilm reactor assessed by changing *D* according to the two strategies (quasi-steadily increase vs. *D*-jumping) pointed out that the performances of the biofilm reactor depended only of the *D* and not on the *D*-tuning strategy.

The last set of experiments was carried out to investigate the effects of the two principal inhibitors: furfural and HMF [25], found in lignocellulosic hydrolysate on the fermentation process. The concentration of the inhibitors in the feeding was set at 1 and 0.28 g L^−1^ [26], for furfural and HMF, respectively. The dilution rate was set at 0.75 and 1.00 h^−1^.

## Results

### Biofilm start-up

**Fig. 2:**
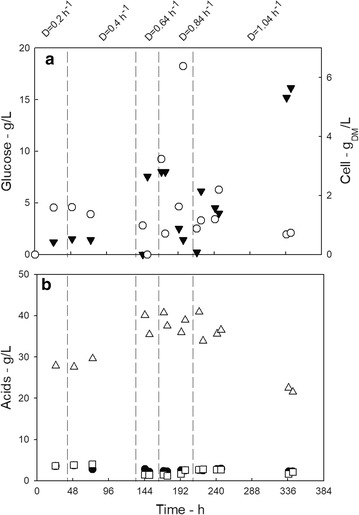
Main data measured during PBBR start-up. The vertical dotted line marks the changes of the dilution rate. **a** Glucose (black down-pointing triangle) and cell concentration (white circle); **b** succinic (white up-pointing triangle), acetic (black circle), and formic (white square) acid concentration

The PBBR was inoculated with actively growing cells at *t* = 0 and operated in batch mode with respect to the liquid phase for 24 h after (data not shown). After 24 h, the PBBR was switched to continuous mode feeding 50 g L^−1^ glucose: medium (synthetic medium), setting that the dilution rate was set at 0.20 h^−1^. A visible biofilm layer formed on the carriers in about 3 days, and at *t* = 7 day, the dilution rate was increased up to 0.84 h^−1^ to promote the biofilm production over the suspended cell growth. Figure 2 reports the time-series of the concentration of acids and glucose during the start-up of the PBBR.

At *t* = 16 days, the carriers were covered with abundant biofilm and steady-state conditions had established in the reactor. Altogether, the biofilm reactor start-up took about 17 days and a significant amount of biofilm was formed. The suspended biomass detected under steady-state conditions was very low and a clear effluent was observed for *D* larger than 0.84 h^−1^.

Provided the stable formation of the biofilm (see Additional file 1: Figure S1), the pH in the reactor was controlled. The pH was set at a value slightly higher than the optimal value reported in literature [27], since a pH and metabolites gradient was expected across the biofilm [16]. As a consequence, the pH within the biofilm was expected to be lower than that measured in the broth.

### Continuous SA production

#### Glucose as carbon source

At *t* = 17 days, the succinic acid production started. The dilution rate was set at 0.5 h^−1^ and the steady state was characterized. The *D* was increased of 0.2 h^−1^ every time to establish a new steady state.

The system approached a steady-state condition within 2–4 days, depending on the dilution rate set. The typical time-series of the fermentation measurements carried out by feeding the glucose-based medium are reported in the Additional file 1: Figure S2. PBBR performance was characterized in terms of metabolite concentration, glucose conversion degree, succinic acid yield, productivity, and selectivity. Data were assessed by processing the dilution rate and the concentration of glucose and metabolites as reported in the “Methods” Section. Data reported in Table 1 were assessed under steady-state conditions: the concentration of the sugar and of the metabolites was constant for at least ten times the reactor mean residence time (*τ* = 1/*D*). The effect of the dilution rate (*D*) on the performance of the PBBR was investigated.

**Table 1:** Biofilm steady-state results measured during fermentation tests carried out with glucose, xylose, and GAX media

Medium	D h^−1^	SA g L^−1^	*ξ*_*S*_%	*Y*_SA/*S*_ g_SA_ g_*S*_^−1^	*χ*_SA_ g g^−1^
Glucose	0.5	43	88	0.98	0.94
	0.64	37.5	81.9	1.03	0.92
	0.74	40.1	77.1	1.02	0.91
	0.8	31	63.2	1.04	0.97
	0.84	31	62.7	0.97	0.91
	0.94	31.3	58.4	1.05	0.88
	1.04	21.5	60.8	0.86	0.85
	1.24	20.1	57.9	0.66	0.83
	1.44	17.6	35	0.96	0.9
	1.54	17.7	38.8	0.93	0.92
	1.7	19.6	37.2	1.05	0.93
	1.9	18.6	34	1.09	0.9
	2.1	14.8	29.8	0.94	0.9
	2.2	13.7	24.9	1.09	0.91
	2.3	11.6	25.3	0.97	0.92
	2.4	9.5	21	0.9	0.92
Xylose	0.5	12.1	37.3	0.81	0.67
	0.6	11	29.8	0.92	0.73
	0.7	8.7	28.8	0.76	0.64
	0.8	8.2	31.1	0.63	0.62
	0.9	8	25	0.8	0.75
	1.04	7.1	19.2	0.93	0.73
	1.24	4.6	16.5	0.7	0.71
	1.44	3.3	2.2	0.67	0.58
GAX	0.7	20.5	45.6	0.56	0.85
	0.75	19.5	46	0.55	0.84
	0.8	17.1	39	0.57	0.87
	1	18	35.5	0.65	0.85
	1.24	12.1	23.5	0.64	0.88
	1.44	11.1	16.2	0.88	0.89

Total sugar concentration: glucose, 50 g L^−1^; xylose, 40 g L^−1^; GAX 80 g L^−1^

**Fig. 3:**
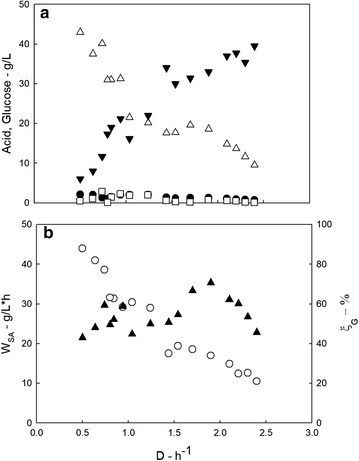
Main data measured during the production phase from glucose-based medium (50 g L^−1^). Data measured during the PBBR operation are reported as a function of the dilution rate; **a** glucose (black down-pointing triangle), succinic (white up-pointing triangle), acetic (black circle), and formic (white square) acid concentration; **b** succinic acid productivity (black up-pointing triangle) and glucose conversion (white circle)

Figure 3 and Table 1 report the main data measured during the continuous fermentation as a function of the dilution rate. The dilution rate was quasi-steadily increased from 0.5 to 2.4 h^−1^. The analysis of the results reported in Fig. 3 and Table 1 highlighted the issues reported hereinafter.

- The glucose conversion degree (*ξ*_G_) and produced succinic acid concentration significantly decreased with *D*. They were characterized by a maximum at the lower investigated *D*.
- Succinic acid productivity was characterized by a maximum (35 g L^−1^ h^−1^) at *D* = 1.9 h^−1^. It is worth to note that SA productivity was the largest values reported in the literature.
- The concentration of acetic and formic acid was always below 5 g L^−1^. Succinic acid selectivity was quite high and about constant with *D* (ranging between 0.84 and 0.96 g g^−1^).

#### Xylose as carbon source

PBBR performance feeding a synthetic medium bearing xylose was investigated, because the xylose represents the main pentose sugar found in lignocellulosic biomass hydrolysate [28, 29]. A test campaign was addressed to adapt the cells to the new sugar: tests with medium containing both glucose and xylose (GX medium) were carried out. The fraction of xylose was progressively increased from 0 to 100% and the dilution rate was set at 1.24 h^−1^. The time-series of the fermentation measurements carried out by feeding the xylose/glucose-based medium are reported in the Additional file 1: Figure S3.

**Fig. 4:**
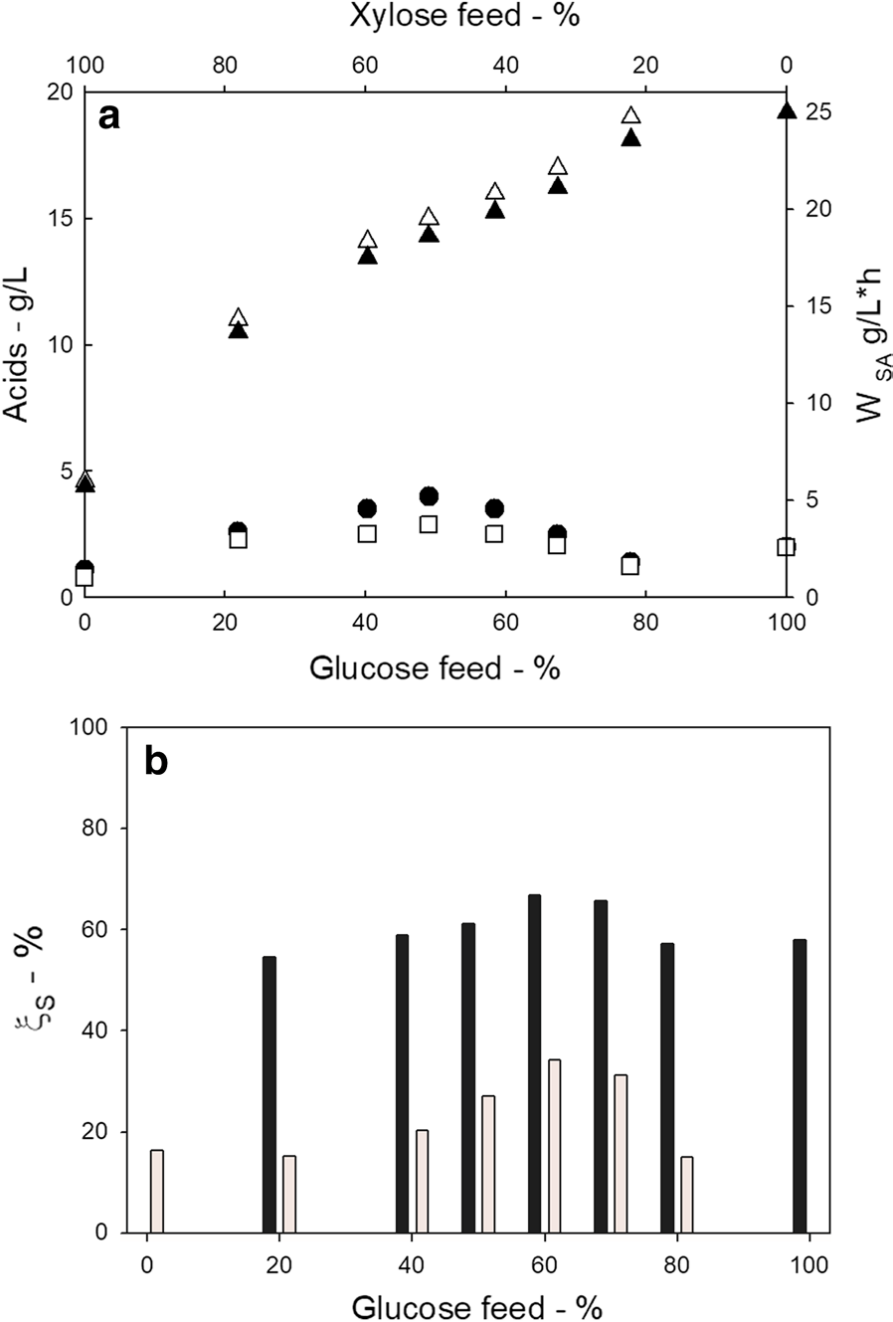
Main data measured during the adaptation phase. Data measured during the PBBR operation as a function of the dilution rate using a glucose-xylose based medium (total sugar concentration 50 g L^−1^). **a** succinic (white up-pointing triangle), acetic (black circle), and formic (white square) acid concentration and succinic acid productivity (black up-pointing triangle); **b** sugars conversion degree (glucose conversion: black bars; xylose conversion: grey bars)

In Fig. 4a, the concentration of the acids and the succinic acid productivity are reported as a function of the sugar composition of the medium (percentage of xylose). Succinic acid concentration and productivity decreased with xylose fraction in the feed. The observed behaviour of SA production is in agreement with the results of tests carried out under batch conditions: *A. succinogenes* metabolizes glucose better than xylose [14]. It interesting to note that increasing the percentage of xylose in the media, acetic and formic acid production also increases and the SA selectivity decreased.

Figure 4b reports sugar conversion as function of percentage of xylose in the feeding. Glucose conversion is constant (around 60%), whereas xylose conversion was characterized by a maximum (35%) when the xylose concentration in the feeding is about 40%.

As the biofilm was adapted to the xylose, the third set of experiments started, feeding the PBBR with a xylose-based medium (xylose concentration set at 40 g L^−1^); the dilution rate was set between 0.5 and 1.44 h^−1^. The steady states were characterized in terms of acids concentration and xylose conversion degree. The typical time-series of the fermentation measurements carried out by feeding the xylose-based medium are reported in the Additional file 1: Figure S4.

**Fig. 5:**
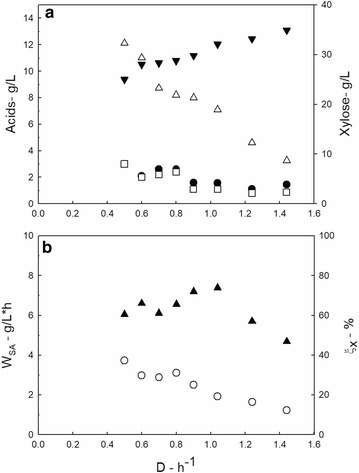
Main data measured during the production phase using a xylose-based medium (40 g L^−1^). Data measured during the PBBR operation as a function of the dilution rate; **a** xylose (black down-pointing triangle), succinic (white up-pointing triangle), acetic (black circle), and formic (white square) acid concentration; **b** succinic acid productivity (black up-pointing triangle) and xylose conversion (white circle)

As reported in Fig. 5, succinic acid concentration and xylose conversion decreased with the dilution rate. These results were expected as an effect of the reduced residence time in the bioreactor.

Succinic acid productivity was quite low when using xylose as the sole carbon source: it reached the maximum value 7.38 g L^−1^ h^−1^ at *D* = 1.04 h^−1^.

#### Glucose–Arabinose–Xylose (GAX) as carbon source

The PBBR performances were evaluated for test carried out feeding the bioreactor with a synthetic lignocellulosic hydrolysate (inhibitor-free) containing glucose, arabinose and xylose. The total sugar concentration in the synthetic medium was set at 80 g L^−1^ and the mass ratio between the sugars was 55:15:30 for the GAX mixture [24]. The dilution rate ranged between 0.7 and 1.44 h^−1^ and each steady-state condition was characterized in terms of acid concentration and sugar conversion degrees. The typical time-series of the fermentation measurements carried out by feeding the GAX-based medium are reported in the Additional file 1: Figure S5.

**Fig. 6:**
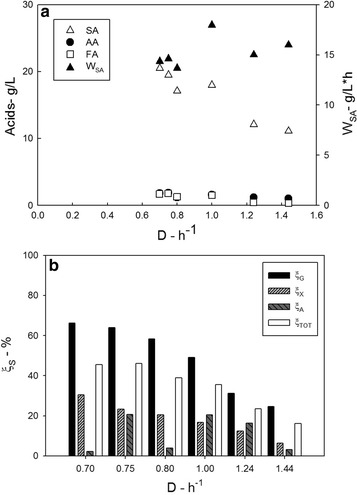
Main data measured during the production phase using the GAX medium. Total sugar concentration: 80 g L^−1^), sugar mass ratio: 55:15:30. **a** succinic (white up-pointing triangle), acetic (black circle), and formic (white square) acid concentration and succinic acid productivity (black up-pointing triangle); **b** sugars conversion (glucose conversion: black bars; xylose conversion: light grey bars; arabinose: dark grey bars total sugars conversion: white bars)

Figure 6a reports that the succinic acid concentration decreased with the dilution rate: the maximum SA concentration was 20.5 g L^−1^ at *D* = 0.7 h^−1^. As regards the sugar conversion degrees (Fig. 6b), glucose and xylose conversion degrees decreased with the *D*, and the arabinose conversion degree varied between 3 and 20%. The total sugar conversion degree also decreased with *D*.

The succinic acid productivity was fairly constant with *D* and it was about 15 g L^−1^ h^−1^.

The PBBR performances expressed in terms of SA concentration and productivity were slightly lower in the tests carried out with GAX solution (Fig. 6) than that of measured in tests with GX solution (Fig. 4): at the same dilution rate (*D* = 1.24 h^−1^) and similar glucose and xylose fraction (about 60% glucose 30% xylose) in the feeding: the SA concentration and productivity decreased when arabinose was present.

#### Effect of inhibitors on succinic acid production

The effect of the potential byproducts of the lignocellulosic hydrolysate—furfural and HMF—typically acting as fermentation inhibitors [25] was investigated. The reactor feeding was supplemented with single potential inhibitors to point out the individual role on the fermentation performance. The succinic acid production by *Actinobacillus* biofilm was characterized during the feeding of a GAX solution supplemented with 1 g L^−1^ furfural and 0.28 g L^−1^ [26], HMF. They were added separately into the GAX medium and their effect was evaluated at two different dilution rates (0.75 and 1.00 h^−1^).

Table 2 reports the main data measured/calculated for the fermentation tests carried out by supplementing furfural and HMF to the GAX medium. The concentration of produced succinic acid decreased with respect to the inhibitor-free medium as inhibitors were supplemented. The SA concentration decreased of about 5.6% and 16% (at *D* = 0.75 and 1.0 h^−1^, respectively) in the presence of furfural, while the concentration of SA produced was reduced by 10.8 and 22.6% when HMF was supplemented, compared to the inhibitor-free GAX medium at the same operating conditions.

**Table 2:** Main results of fermentation test adding furfural or HMF to the GAX medium, compared to the inhibitor-free GAX tests

D—h^−1^	0.75			1.00		
	*GAX*	*GAX *+* Furfural*	*GAX *+* HMF*	*GAX*	*GAX *+* Furfural*	*GAX *+* HMF*
SA— g L^−1^	19.50	18.40	17.40	17.96	15.10	13.90
*ξ*_TOT_—%	46.00	50.97	43.40	35.45	35.83	35.21
W_SA_— g L^−1^h^−1^	14.60	13.80	13.05	17.96	15.10	13.90
Y_SA_—g_SA_ g_S_^−1^	0.55	0.50	0.56	0.65	0.58	0.55
χ_SA_ g_SA_ g_TotAc_^−1^	0.84	0.75	0.84	0.85	0.86	0.87

The SA selectivity was not affected by the presence of the inhibitors. This result would suggest that the flux distribution between the C4 (metabolic pathway leading to SA production) and the C3 pathway (metabolic pathway leading to AA and FA production) [30] does not change as inhibitors are present in the medium.

The biofilm PBBR was stopped at the end of the run with the inhibitors, after 5 months of continuous operation and the overall biomass concentration was 107 g_DM_ L^−1^.

## Discussion

The biorefinery approach to produce bio-based products may become competitive with respect to the currently used petroleum route, because the depletion of fossil resources and the optimization of biotechnological processes are in progress. Various types of chemicals that are conventionally produced by chemical processes could potentially be generated via biotechnological processes using biological materials as feedstocks and microorganisms as biocatalysts [31].

Considering that, for bulk chemicals to be successful on the market their price needs to be low, investments in equipments as well as the operating costs of the industrial production process also need to be extremely low. The cost of the fermentation substrate is one of the key features for an economically viable process. The use of agriculture and forest-related residues, industrial waste, and by-product streams has a high potential as an alternative and sustainable source of raw material for chemical industries.

Succinic acid is well established as bio-based platform chemical with production quantities expecting to increase exponentially within the next decade. *Actinobacillus succinogenes* is by far the most studied wild-type succinic acid producing microorganism and the most interesting for industrial applications. Being facultative anaerobic microorganisms, its use in a bio-based process for SA production would reduce the bioreactor costs due to the absence of aeration that increases significantly capital and operating costs.

Despite the requirement for high productivities to reduce the production costs, the majority of the literature publications are focused on batch fermenters, typically characterized by low productivity and long dead time. From a processing perspective, high cell density fermentation could enhance volumetric productivity and reduce capital costs. Given the economic requirement for high cell density fermentation, more insight is required on the rate and yield characteristics of *A. succinogenes* biofilms.

In the present study, a packed-bed biofilm reactor was developed for succinic acid production by *A. succinogenes* from a synthetic lignocellulosic biomass hydrolysate.

The results reported in the present study are very promising when compared to the previous investigation regarding SA production by *A. succinogenes*. In particular, in Corona-González et al. [32], the production of succinic acid with *A. succinogenes* entrapped in agar beads was studied. The succinic acid concentration of 43.4 g/L was obtained from 78 g L^−1^ glucose, corresponding to a volumetric productivity of 0.68 g L^−1^ h^−1^. Continuous anaerobic fermentations in a biofilm reactor packed with Poraver^®^ beads were also carried out by Maharaj et al. [33]. They reported a volumetric productivity of 10.8 g L^−1^ h^−1^ at *D* = 0.7 h^−1^ using a glucose-based medium. The highest productivity reported in the literature was obtained by Brink and Nicol [34]. They obtained a productivity of 17.1 g L^−1^ h^−1^ at *D* = 2.2 h^−1^ using a novel shear-controlled fermenter, that enabled both chemostat and biofilm operation.

In the present investigation, was obtained the highest productivity among that reported in the literature: 35.0 g L^−1^ h^−1^ for glucose fermentation. The optimal results were obtained at the dilution rate 0.5 h^−1^: 43.0 g L^−1^ of succinic acid were produced, glucose conversion was 88%; and the volumetric productivity was 22 g L^−1^ h^−1^, still higher than that reported in the literature.

Succinic acid productivity was much lower when feeding the PBBR with xylose as the sole carbon source: the maximum productivity was 7.38 g L^−1^ h^−1^ at *D* = 1.04 h^−1^. However, the productivity is still interesting when compared with results available in the literature. Bradfield and Nicol [35] reported succinic acid production from pure xylose by *A. succinogenes* biofilm and they found that the production was lower than 4 g L^−1^ h^−1^ at the three investigated dilution rate (*D* = 0.05, 0.10, and 0.30 h^−1^).

The possibility to produce succinic acid from different sugars by *A. succinogenes* is in agreement with the previous investigations [14]: the bacterium could simultaneously uptake glucose, mannose, arabinose, and xylose to produce succinic acid. Therefore, it was expected to have the co-fermentation of the sugar present in the synthetic lignocellulosic hydrolysate medium (GAX medium) by *A. succinogenes* biofilm.

It is worth to note to compare the results reported in the present investigation with those reported by Bradfield et al. [36]. They used a custom continuous fermentation setup to produce SA from corn stover hydrolysate stream, containing xylose, glucose, arabinose, and galactose, produced from deacetylation and dilute acid pretreatment. The maximum SA concentration, yield, and productivity were 39.6 g L^−1^, 0.78 g g^−1^, and 1.77 g L^−1^ h^−1^, respectively, at a dilution rate of 0.05 h^−1^. Despite the low SA concentration measured during the tests carried out with the GAX solution (Fig. 6), the productivity was almost one order of magnitude larger than that reported by Salvachúa et al. [37]. To the author knowledge, this is the only study available in the scientific literature regarding the continuous SA production from lignocellulosic hydrolysate by *Actinobacillus* biofilm. Further comparison with SA production studies using *A. succinogenes* and biomass **feedstocks** may be proposed only with reference to batch fermentation mode.

The effect of the primary suspected fermentation inhibitors, furfural and HMF, was also investigated in this study. It was found that both the inhibitors reduced succinic acid production when compared with results of the tests with an inhibitor-free GAX medium. The HMF had a stronger inhibiting effect compared to furfural. However, it should be pointed out that the concentration of the inhibitor species was not monitored during the tests; therefore, it is not known if there was any conversion of the inhibitors. As suggested in a previous study [38], furfural can be converted to furfuryl alcohol by means of an aldehyde reductase, because the aldehyde may be reduced to its alcohol form. Moreover, the genome of *A. succinogenes* encodes an aldo/keto reductase [39] that may be responsible for the reduction of furfural.

## Conclusions

Succinic acid production by *A. succinogenes* fermentation in a packed-bed reactor (PBBR) was successfully carried out for more than 5 months. The effects of the dilution rate (D) and medium composition (glucose, GX, xylose, and GAX media) on the PBBR performances were investigated. Succinic acid concentration, productivity, and sugar(s) conversion generally decreased with D. A maximum succinic acid productivity of 35.0 g L^−1^ h^−1^ was achieved at *D* = 1.9 h^−1^. The effect of two inhibitors was also investigated. HMF remarkably reduced succinic acid production when compared to furfural.

## Additional file

**Additional file 1: Figure S1.** Biofilm of *A. succinogenes.* a) at the end of the start-up phase; b) after 5 months of continuous operation. **Figure S2.** Time-course profiles of the fermentation results during the production phase from glucose. **a** Glucose (▼) and cell concentration (○) and dilution rate (dashed line); **b** succinic (∆), acetic (●) and formic (□) acid concentration and dilution rate. **Figure S3.** Time-course profiles of the fermentation results during the adaptation phase from glucose to xylose. **a** Glucose (▼), xylose (■) and cell concentration (○); **b** succinic (∆), acetic (●), and formic (□) acid concentration and xylose percentage (dashed line) in the medium. The dilution rate was set to 1.24 h^−1^. **Figure S4.** Time-course profiles of the fermentation results during the production phase from xylose. **a** Xylose (▼) and cell concentration (○) and dilution rate (dashed line); **b** succinic (∆), acetic (●), and formic (□) acid concentration and dilution rate. **Figure S5.** Time-course profiles of the fermentation results during the production phase from GAX medium. **a** Sugars [glucose (▼), xylose (■), and arabinose (∇)] and cell concentration (○) and dilution rate (dashed line); **b** succinic (∆), acetic (●), and formic (□) acid concentration and dilution rate.

## Abbreviations

AA: acetic acid
BHI: brain heart infusion broth
D: dilution rate
FA: formic acid
GAX: glucose–arabinose–xylose medium
GX: glucose–xylose medium
HMF: hydroxymethyl furfural
HPLC: high-pressure liquid chromatography
ID: internal diameter
OD: optical density
PBBR: packed-bed biofilm reactor
SA: succinic acid
TCA: tricarboxylic acid cycle
