# Supplementary material for: Continuous succinic acid fermentation by Actinobacillus succinogenes in a packed-bed biofilm reactor
Source: Biotechnol Biofuels. 2018 May 14;11:138. doi: 10.1186/s13068-018-1143-7 (PMC5950251; doi:10.1186/s13068-018-1143-7)
Supplement: Supplementary file 1 — Additional file 1: Figure S1. Biofilm of A. succinogenes. a) at the end of the start-up phase; b) after 5 months of continuous operation. Figure S2. Time-course profiles of the fermentation results during the production phase from glucose. a Glucose (▼) and cell concentration (○) and dilution rate (dashed line); b succinic (∆), acetic (●) and formic (□) acid concentration and dilution rate. Figure S3. Time-course profiles of the fermentation results during the adaptation phase from glucose to xylose. a Glucose (▼), xylose (■) and cell concentration (○); b succinic (∆), acetic (●), and formic (□) acid concentration and xylose percentage (dashed line) in the medium. The dilution rate was set to 1.24 h−1. Figure S4. Time-course profiles of the fermentation results during the production phase from xylose. a Xylose (▼) and cell concentration (○) and dilution rate (dashed line); b succinic (∆), acetic (●), and formic (□) acid concentration and dilution rate. Figure S5. Time-course profiles of the fermentation results during the production phase from GAX medium. a Sugars [glucose (▼), xylose (■), and arabinose (∇)] and cell concentration (○) and dilution rate (dashed line); b succinic (∆), acetic (●), and formic (□) acid concentration and dilution rate. [file 13068_2018_1143_MOESM1_ESM.docx]

**Supplementary material**

*
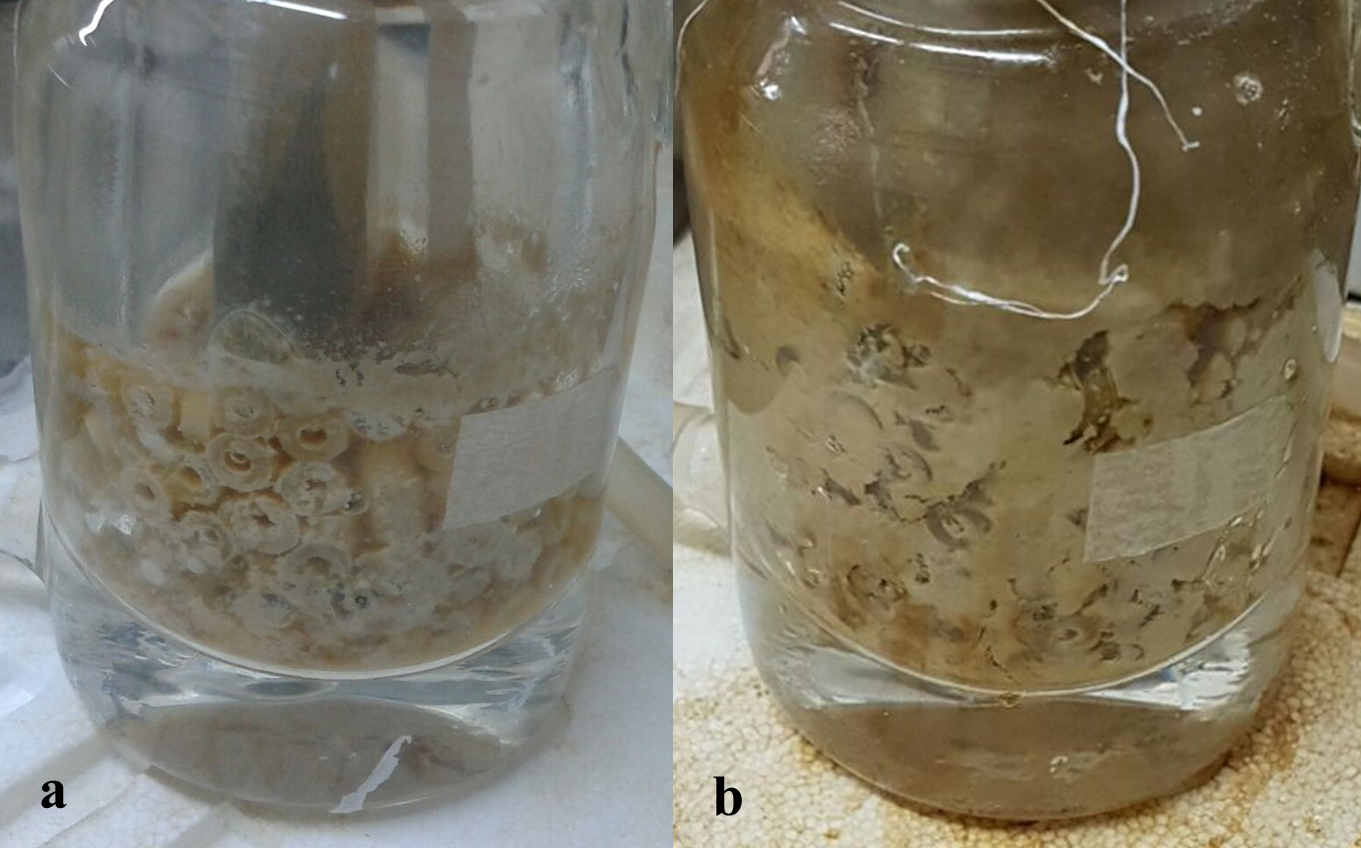
*

**Figure S1 –** Biofilm of *A. succinogenes.* a) at the end of the start-up phase; b) after 5 months of continuous operation.


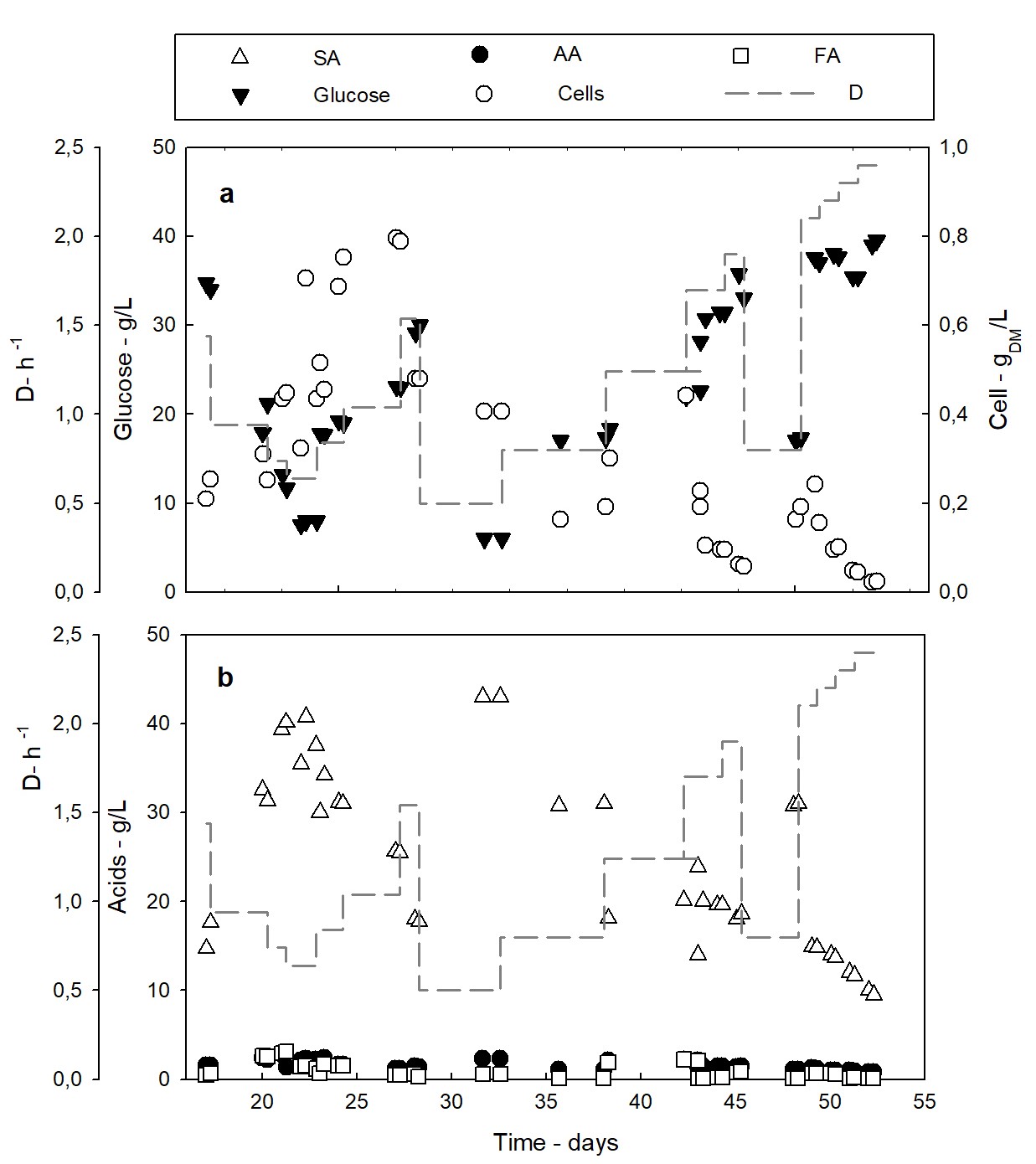


**Figure S2–** Time-course profiles of the fermentation results during the production phase from glucose. **a**. glucose (▼) and cell concentration (🞅) and dilution rate (dashed line); **b**. succinic (△) , acetic (●) and formic (□) acid concentration and dilution rate.

**
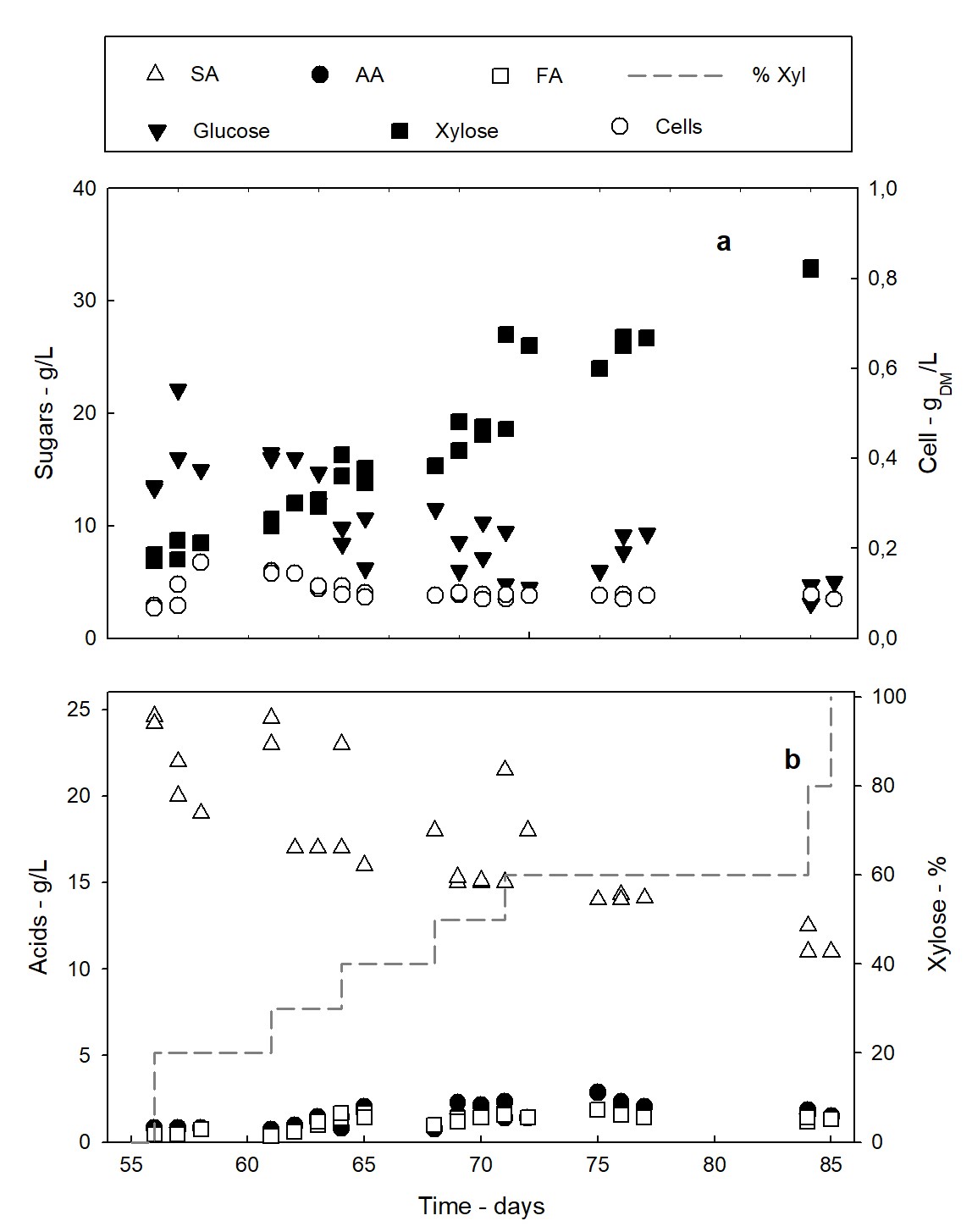
**

**Figure S3–** Time-course profiles of the fermentation results during the adaptation phase from glucose to xylose. **a**. glucose (▼), xylose (■) and cell concentration (🞅); **b**. succinic (△),acetic (●) and formic (□) acid concentration and xylose percentage (dashed line) in the medium. The dilution rate was set to 1.24 h^-1^.


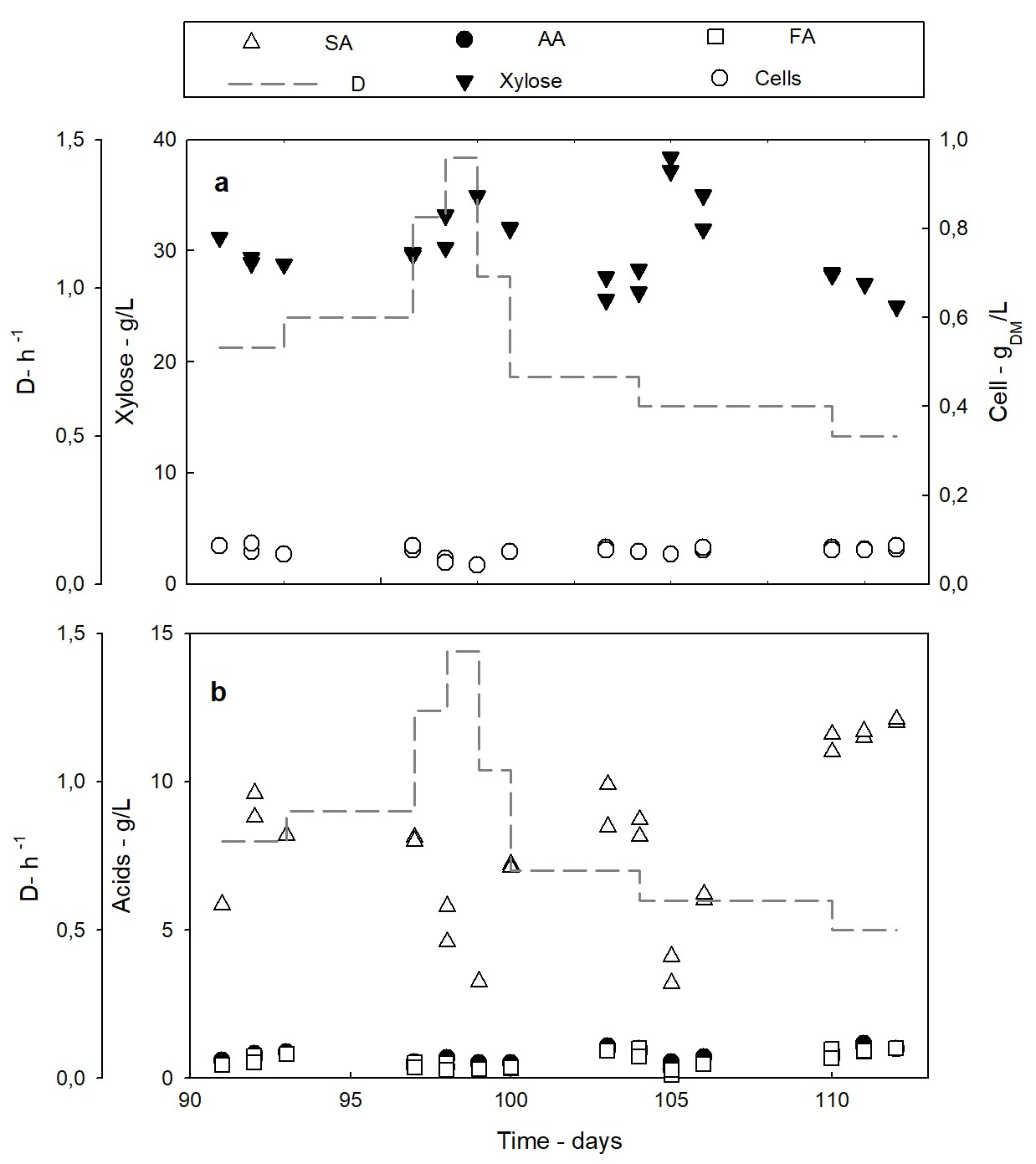


**Figure S4–** Time-course profiles of the fermentation results during the production phase from xylose. **a**. xylose (▼) and cell concentration (🞅) and dilution rate (dashed line); **b**. succinic (△) , acetic (●) and formic (□) acid concentration and dilution rate.


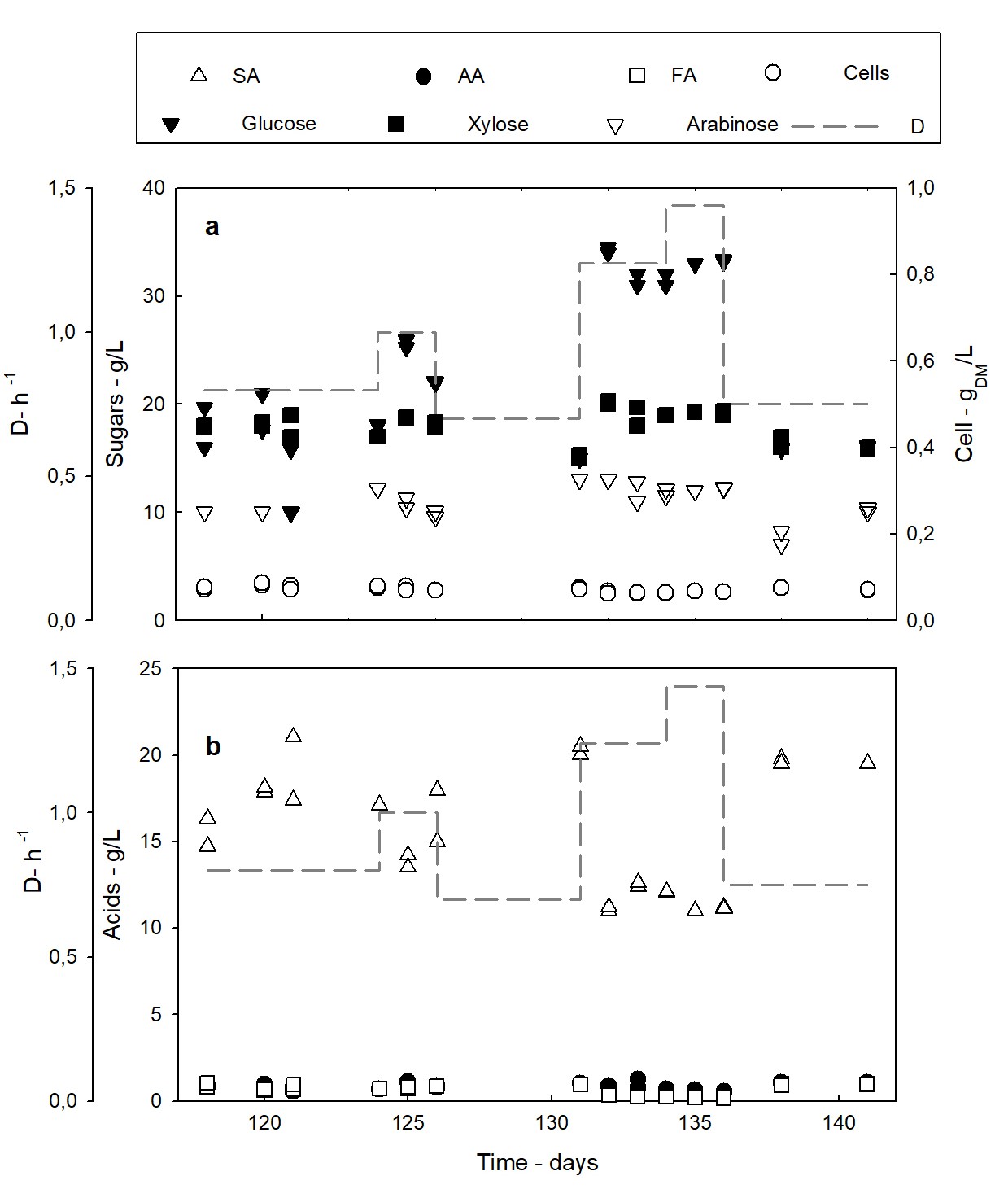


**Figure S5 –** Time-course profiles of the fermentation results during the production phase from GAX medium. **a**. sugars (glucose (▼), xylose(■) and arabinose (∇)) and cell concentration (🞅) and dilution rate (dashed line); **b**. succinic (△) , acetic (●) and formic (□) acid concentration and dilution rate.
